# Vascular Endothelial Protection Effects of *Curcuma wenyujin* Root Aqueous Extracts on LPS-induced Rat Vascular Damage

**DOI:** 10.7150/ijms.112103

**Published:** 2025-07-28

**Authors:** Li-Hsien Chen, Sheau-Chung Tang, Shu-Er Yang, Erl-Shyh Kao

**Affiliations:** 1 Department of Food Science, Tunghai University, Taichung 407224, Taiwan.; 2 Department of Nursing, National Taichung University of Science and Technology, Taichung 403027, Taiwan.; 3 Department of Beauty Science and Graduate Institute of Beauty Science Technology, Chienkuo Technology University, Changhua 500020, Taiwan.; 4 Department of Beauty Science, National Taichung University of Science and Technology, Taichung 403027, Taiwan.

**Keywords:** lipopolysaccharides, acute vascular damage, matrix metalloproteinase, *Curcuma* wenyujin

## Abstract

Sepsis caused by bacterial infection can also induce vascular endothelial damage through endotoxins secreted by bacteria such as lipopolysaccharides (LPS). The mechanism of LPS induce vascular endothelial damage is mainly through the release of pro-inflammatory factors and activation of matrix metalloproteinases (MMPs), then MMPs further cause the glycocalyx layer to damage endothelial cells and finally lead to hyperosmolar vascular abnormalities and eventually microcirculatory disorders in general clinical practice, and are used in clinical treatment to prevent the release of pro-inflammatory factors. However, the immunosuppressive effect of high-dose dexamethasone is unpredictable before pathogens are cleared. *Curcuma wenyujin* (CW) is a traditional Chinese medicine containing the biologically active ingredient β-elemene, which has been reported to have endothelial protective effects. In this study, an acute vascular endothelial damage animal model was established by intraperitoneal injection of LPS in rats, and the treatments included oral administration of CW root aqueous extract solution at a low dose (LW group, 375 mg/kg/day) and high dose (HW group, 1500 mg/kg) for endothelial protection evaluation. The results demonstrated that HW reduced the TLR4 signaling pathway and downstream markers of vascular inflammation, particularly MMP2 and MMP9. This study suggests that CW, a traditional Chinese medicine, could CW root aqueous extracts treatments protect against LPS-induced acute vascular damage in rats. This study advocates for further clinical exploration of CW as a potential clinical use in bacterial infection-induced sepsis or complementary treatment to current therapies, potentially benefiting cardiovascular and other inflammatory conditions.

## Introduction

Bacteria or viral infections are the major pathogens causing sepsis, and at present, antibiotics are used for infection source control or organ function support 1. Bacteria are easily identified and eliminated by antibiotics in bacteremia, but the exotoxins, endotoxins, and other microbial products will continue the development of sepsis in the host into different types, including fulminant, acute, subacute, and chronic 2. Eliminate of bacteria is a major process in sepsis treatment, and the prevention of vessel or organ damage and restoration of homeostasis, immune reactions, and dynamic balance are more important. Vascular damage, caused by endotoxin-induced glycocalyx damage, is the major cause of sepsis and leads to organ dysfunction 3.

Endotoxins, also called lipopolysaccharides (LPS), are recognized by Toll-like receptor 4 (TLR4) and trigger downstream signaling in endothelial damage responses 4. The combination of TLR4 and LPS induces downstream nuclear factor kappa B (NF-κB) through the MyD88 (myeloid differentiation factor 88) signaling pathway, thereby enhancing the expression of inflammatory factors 5. Although the main purpose of TLR4 activation is immune defence, the inflammatory response it triggers may also cause tissue damage 6. Sustained or excessive TLR4 activation leads to continuous release of inflammatory mediators, thereby promoting tissue damage and cell apoptosis 7. In addition, the inflammatory environment after TLR4 activation can indirectly trigger the production of tissue-degrading enzymes, such as MMPs 8.

Deglycosylation refers to the removal of sugar groups from proteins by enzymes 9. This change affects the stability and function of proteins 10. Deglycosylation may be a key factor in promoting the production of MMPs in the inflammatory environment activated by TLR4 11. Deglycosylation can increase the transcriptional activity of MMPs, thereby increasing their expression. In particular, MMP2 and MMP9 can degrade the collagen structure in the ECM, leading to tissue structure instability 12. Under normal conditions, MMPs play regulatory roles in tissue remodeling and repair 13. However, under deglycosylation induction, overexpression of MMPs leads to excessive degradation of ECM structures, thereby damaging tissue integrity and function 14. Especially in chronic inflammatory environments, overexpression of MMPs can accelerate cell death and irreversible damage 15.

Endothelial cells are located in the inner layer of blood vessels and play an important role in maintaining normal blood vessel function, regulating blood pressure, and regulating inflammatory responses 16. However, inflammatory responses often lead to endothelial cell damage, which in turn leads to vasculopathy and various cardiovascular diseases 17. As a natural hormone, E2 estrogen or phytoestrogen reportedly inhibits the activation of NF-κB, reduces the production of inflammatory factors, reduces endothelial cell protection, and inhibits inflammatory responses 18, 19. In this context, *Curcuma wenyujin* (CW) is a traditional Chinese medicine containing the biologically active ingredient β-elemene, which was reported to exert endothelial protection effects 20. β-Elemene is a phytoestrogen compound with estrogenic characteristics and a similar chemical structure as estrogen. This compound has been predicted to protect endothelial cells from inflammatory damage 21.

In this study, different doses of CW root aqueous extract pretreatment and LPS were used to induce vascular endothelial damage in SD rat models. The results showed that CW in the high-concentration treatment group exerted vascular endothelial protective effects through TLR4 and its downstream information pathway inhibition.

## Materials and Methods

### Preparation of extracts

The CW root aqueous extract was freshly prepared before use. CW root slices (400 g) were soaked in 1000 mL distilled water and refluxed for 1 h for extraction. After cooling, the CW root aqueous extract solution was filtered through Whatman grade 4 filter paper (Sigma-Aldrich, St Louis, MO, USA). The filtered solution contained 375 mg/mL CW total extract.

### High performance liquid chromatography

The major components of the CW aqueous extract and β-elemene (30 mM) standard solutions were analyzed by HPLC. One milliliter of CW aqueous extract or β-elemene (30 mM) standard solution was centrifuged at 10,000 × *g* for 30 min, 4℃ and the supernatant was collected and filtered through 0.22 μm filter and immediately analyzed by HPLC. The condition of the mobile phase (pH = 3.5) was a mixture solution composed by 69 % of 60 mM phosphoric acid solution and 31% acetonitrile in v/v). The flow rate setting of the HPLC was 1 mL/min using a 150×4.6 mm C-18 column (RP-18 GP, Kanto Mightysil, Japan), and the 350 nm absorbance was measured using a UV detector (SPD-20A, Shimazu, Japan).

### Animals

Total 96 SD male rats (250 ± 9 g) were purchased from BioLASCO Taiwan Co., Ltd. The animal use protocol was reviewed and approved by the Institutional Animal Care and Use Committee or Panel (IACUC/ IACUP) of Tunghai University (110-034). In this experiment, the animal standard living conditions were 25°C, 60% humidity and a 12 h light/dark cycle. All SD rats were fed a normal diet (Laboratory Rodent Diet 5001) and provided water* ad libitum*. The experiment was initiated and all rats were randomized into four groups (n=12) after 1 week of acclimation. Group 1 (control) was designated as the control group, and rats in this group were treated with normal saline (1 mL) through gavage once a day in the morning for 3 days and then intraperitoneally (IP) injected with normal saline (1 mL) on the third day after gavage treatment. Group 2 (LPS) was designated as the LSP-induced vascular damage group, and rats in this group were treated with normal saline (1 mL) through gavage once a day in the morning for 3 days and then intraperitoneally (IP) injected with LPS (15 mg/ml in PBS) on the third day after gavage treatment. Group 3 (LW) was designated as a low-dose CW treatment group, and rats in this group were treated with CW root aqueous extract solution (375 mg/kg) through gavage once a day in the morning for 3 days and then intraperitoneally (IP) injected with LPS (15 mg/mL in PBS) on the third day after gavage treatment. Group 4 (HW) was designed as a high-dose CW treatment group, and rats in this group were treated with CW root aqueous extract solution (1500 mg/kg) through gavage once a day in the morning for 3 days and then intraperitoneally (IP) injected with LSP (15 mg/mL in PBS) on the third day after gavage treatment. All rats were sacrificed, and aortic tissues were collected 6 h after LPS IP injection.

### Pathology

The aortic tissues were washed twice with PBS and then soaked in 3.9% formalin solution. On the second day, the tissues were dehydrated using alcohol solutions and embedded in paraffin wax. Then, 5 μm-thick paraffin slices were prepared from the paraffin-embedded tissue blocks. All slices were deparaffinized in xylene and then rehydrated in alcohol solutions from 90% decrease to 60%. The tissue slices were stained with hematoxylin-eosin, and the resulting images were scanned using the P250 FLASH system (3DHISTECH, Ltd. Budapest, Hungary) and imaged using CaseViewer v2.1 edition software (3DHISTECH, Ltd., Budapest, Hungary).

### Protein sampling and analysis

All rat abdominal aorta tissues were collected and homogenized with a protein extraction solution (PRO-PREPTM, iNtRON Biotechnology, Korea) on ice. The supernatant of each sample was collected after centrifugation and the protein concentration was measured and diluted to 10 mg/mL. The protein expression level of each sample was analyzed by western blotting. The standard operation protocol is using electrophoresis to separate the target proteins by 12% SDS-PAGE and then transferred to polyvinylidene difluoride membranes (Hybond-C, GE Healthcare UK, Ltd., Little Chalfont, UK). Before the primary antibody recognition, the 5 % BSA Tris-buffered saline solution was used for blocking assay. The primary antibodies used in this study were TLR4 (ab217274, Abcam, Cambridge, UK), MyD88 (#4283, Cell Signaling Technology, MA, USA), and IRAK1 (ab238, Abcam Cambridge, UK). IRAK4 (#4363, Cell Signaling Technology, MA, USA), p-TAK1 (#9339, Cell Signaling Technology), TAK1 (#5206, Cell Signaling Technology), NF-κB (#8242, Cell Signaling Technology), MMP2 (#40994, Cell Signaling Technology), MMP9 (ab228402, Abcam, Cambridge, UK), and GAPDH (#5174, Cell Signaling Technology). The secondary antibody used was anti-rabbit IgG (#7074; Cell Signaling Technology, MA, USAUSA). The protein expression levels presented on the membranes were visualized using an imaging system (ChemiDoc BioRad, California, USA).

### Statistical analysis

Statistical analysis was performed using one-way analysis of variance (ANOVA) for indicated paired groups, and all results are presented as mean ± SD. A * symbol labeled on the result presented *p*-value less than 0.05, and was considered statistically significant and labeled with. The statistical analysis software used was SigmaPlot v.10.0.

## Results

### HPLC anaylsis results

**Figure 1:**
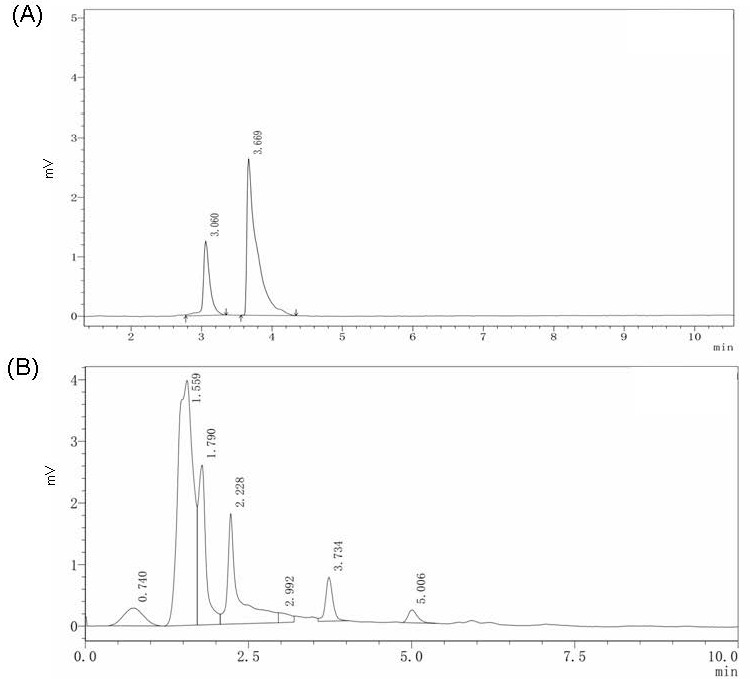
HPLC analysis results. (A) The 30 mM β-elemene standard solution HPLC analysis result and the retention time of β-elemene is 3.669 min. (B) The HPLC analysis result of CW extract solution the retention time of β-elemene is 3.734 min.

HPLC analysis of β-elemene (30 mM) standard solution showed that the retention time (Rt) was 3.669 min and peak area was 25,443 units (Figure 1A). A peak for β-elemene peak was observed at 2,460 units (Figure 1B). Further calculations indicated that 100 mg of CW extract contains 25.7 μg β-elemene. The concentration of β-elemene administrated to the cells and animal models reported in a previous study was 100 mg/L 22. In our study, the low-dose CW group received 375 mg/kg/L (containing 96.4 mg/L β-elemene) and high-dose CW received 1500 mg/kg (containing 385.6 mg/L β-elemene).

### CW pretreatment protects against LPS-induced vascular damage effects

**Figure 2:**
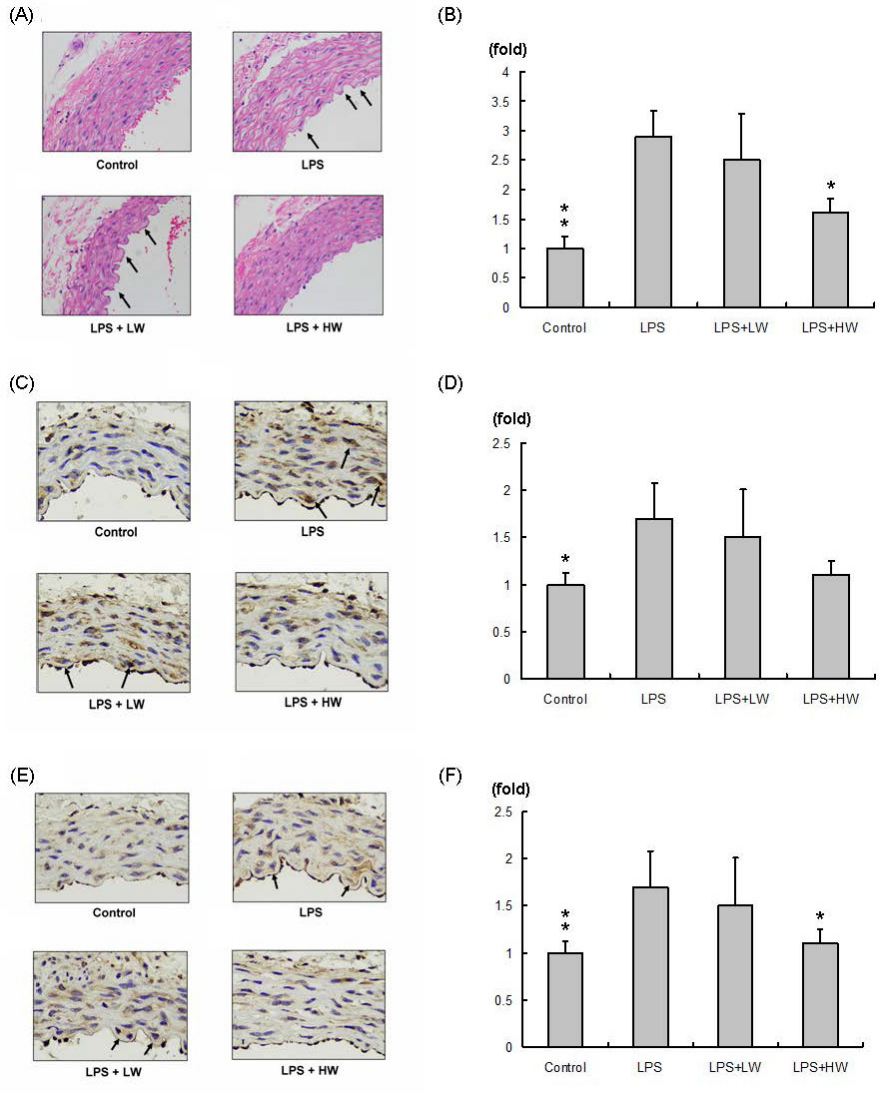
Immunohistochemical staining assay results. (A) Hematoxylin and eosin (H&E) staining of vascular endothelial tissue from each group; swollen vascular endothelial tissue is indicated with an arrow. (B) Relative swelling area in each group normalized to the control group. (C) MMP2 staining in each group. (D) MMP2 expression normalized to the control group. (E) MMP9 staining in each group. (F) MMP9 expression normalized to the control group. (*P < 0.05, **P < 0.01, ***P < 0.001 compared with the LPS treatment only group).

The hematoxylin and eosin-stained vascular tissue sections (100× significant) are shown in Figure 2A. Compared with the control group, the LPS and vascular damage groups showed more obvious swelling (indicated by an arrow). Swelling of vascular tissue sections in the LW-treated group did not change, whereas that in the HW-treated group was significantly decreased and was similar to that in the control group. The related swelling area normalized to control group is presented in Figure 2B. Compared with the LPS treatment group, the LW-treated group exhibited no specifically change, whereas the HW-treated group showed significantly reduced swelling (*P* < 0.01).

### CW pretreatment reduced MMPs in LPS-induced vascular damage

MMP2 expression in each vascular tissue slice (200× significant) is presented in Figure 2C; the results normalized to the control group are shown in Figure 2D. MMP2 expression was increased in the LPS-induced vascular damage group compared to that in the control group. MMP2 was highly expressed in the LW group but slightly reduced in the HW treatment group. MMP9 expression in each vascular tissue slice (200× significant) is presented in Figure 2E; the results normalized to the control group are shown in Figure 2F. MMP9 expression was increased in the LPS-induced vascular damage group compared to that in the control group. MMP9 was highly expressed in the LW group but significantly reduced in the HW treatment groups (*P* < 0.01).

### CW vascular protection effects through MMPs expression level reduction

**Figure 3:**
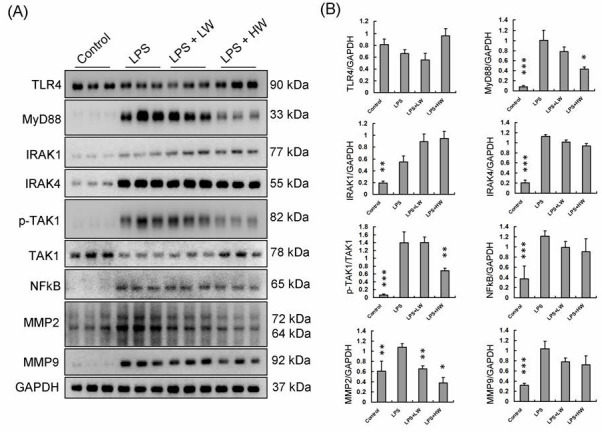
The TLR4 signaling pathway expressions in each group tissue sample. (A) Each proteins expression of indicated treatment groups. (B) Each proteins expression and calibration results. (* means p < 0.05, ** means p < 0.01, ***means p < 0.001 compared with LPS treatment only group).

Western blot protein analysis was performed to determine protein expression levels in rat vascular tissue samples from each group, as shown in Figure 3A. The expression levels of proteins related to the TLR4 signaling pathway were normalized to GAPDH or the original types and presented as fold-changes (Figure 3B). TLR4 expression did not differ between groups. However, MyD88 and its downstream signaling pathway proteins, including IRAK1, IRAK4, NF-κB, MMP2, and MMP9, were increased in the LPS treatment group. In the HW pretreatment group, MyD88 and its downstream signaling pathway proteins showed significantly decreased expression levels (*P* < 0.01).

## Discussion

Vascular endothelial damage caused by LPS protection by CW extract pre-treatment in an animal model was evaluated in this study. CW extract contains the active ingredient, β-elemene, which has potential endothelial protective effects. HPLC analysis of the β-elemene standard and CW root aqueous extract was performed prior to this study (Figure 1). Therefore, when animals were given CW extract treatment, it was already known that the low-dose CW root aqueous extract contained the equivalent of 96.4 μg β-elemene, the high-dose CW root aqueous extract contained the equivalent of 385.5 μg β-elemene.

Multiple studies have shown that β-elemene has anticancer, anti-inflammatory, and immunomodulatory effects, especially in endothelial cell protection and inhibition of inflammation 21. This study reviews the protective mechanism of β-elemene on endothelial cells and the potential mechanism of its anti-inflammatory effect and explores its prospects in clinical applications 22. β-elemene has antioxidant properties and can protect endothelial cells by alleviating oxidative stress by reducing the production of reactive oxygen species (ROS) 23. Studies have shown that β-elemene can activate the nuclear factor red type 2-related factor 2 (Nrf2) signaling pathway, inducing an increase in the expression of antioxidant enzymes (such as superoxide dismutase and glutathione peroxidase), thereby strengthening endothelial cells 24. has been reported to inhibit the NF-κB and JAK/STAT signaling pathways, thereby reducing the production of inflammatory mediators, reducing the release of inflammatory factors, and reducing the damage caused by the inflammatory response of endothelial cells 24.

The study also conducted histopathology, protein detection, and statistical analysis, showing that HW reduced the expression of MMP2 (Figure 2C and 2D) and significantly reduced MMP9 (Figure 2E and 2F) and reduced LPS-induced vascular tissue edema. This study revealed the potential of CW to inhibit inflammatory responses and protect the vascular endothelium, providing new treatment ideas for sepsis-related vascular injury.

Moreover, protein expression in tissue samples from each group (Figure 3) revealed that CW pretreatment inhibited the MyD88-mediated NF-κB pathway, which is an important pathway in the inflammatory response, by inhibiting TLR4 signaling 25. The activation of TLR4 produces a large amount of pro-inflammatory cytokines and induces a series of immune responses 26. When deglycosylation is accompanied by the activation of TLR4, the synergistic effect of the two may significantly enhance the expression of MMPs and form a feedback loop 27. The positive feedback mechanism formed by this interaction has been observed in a variety of chronic diseases, including rheumatoid arthritis, atherosclerosis, and pulmonary fibrosis 28. Interestingly, CW root aqueous extract pretreatment reduced MMP9 expression in HW group tissue slices (Figure 2E and 2F), but the protein analysis results showed that MMP9 expression was not reduced. This result suggests that MMP9 expression may occur not only in the endothelial tissue but also in the tunica intima, unica media, and adventitia or tunica externa 29.

Estrogen (17β-estrogen) exerts a significant protective effect on endothelial cells through multiple mechanisms, including antioxidant and anti-apoptotic activities, promotion of NO production, and inhibition of the inflammatory response 30. Based on these effects, 17β-estrogen has broad application prospects in the prevention and treatment of cardiovascular diseases 31. However, further clinical studies are needed to determine the dose, administration method, and long-term effects of 17β-estrogen to optimize its clinical application and reduce potential risks. Oral 17β-estrogen is rapidly metabolized into other structures and loses its original function 32. The biggest difference between phytoestrogen and 17β-estrogen is that it is already very commonly used as a food supplement, or a common active ingredient in traditional Chinese medicine, such as β-elemene, the main active ingredient of CW used in this study. β-elemene is also a phytoestrogen molecule 21. β-Elemene as well as other compounds are responsible for the effects of CW extract. However, HPLC analysis can typically only be performed using individual standards, and β-elemene is the most abundant compound in CW extracts. Analysis of other present in CW extract requires the use of different HPLC detectors, separation columns, and infusion conditions. Based on the limited evidence from this study, only β-elemene can be discussed.

**Figure 4:**
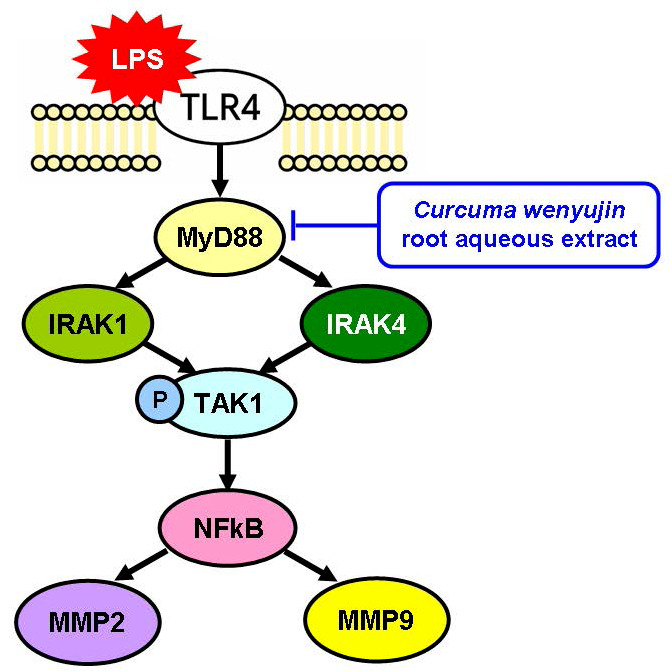
*Curcuma wenyujin* root aqueous extract inhibits the LPS-TLR4-MyD88 signaling pathway.

Treatment with β-elemene can reduce the expression of TLR4, NF-κB, and MyD88 but not significant in an LPS-stimulated group 33. In our study, pretreatment with CW extract reduced LPS-induced TLR4-MyD88 signaling and downstream expression of NF-κB, MMP2, and MMP9 (Figure 4). In clinical use, the dosage can be adjusted with reference to the dosage used in traditional Chinese medicine, or the main active ingredients can be further purified, and then more precise pharmacokinetic experiments can be conducted.

In conclusion, pretreatment with CW root aqueous extract can be used to prevent and reduce vascular damage caused by sepsis, which provides a new perspective on the application of traditional Chinese medicine in modern medicine. Patients with sepsis often develop multiple organ dysfunction due to endothelial damage caused by the over-activation of the immune system. Current treatments mainly rely on antibiotics to control the source of infection and anti-inflammatory drugs to control the inflammatory response 34. However, long-term use of anti-inflammatory drugs may cause adverse side effects, especially immunosuppressive effects, which may cause risks before the pathogen is eliminated 35. In contrast, CW, as a natural botanical medicine, has few toxic side effects and protects endothelial cells by regulating immune responses at multiple levels, which has potential clinical application value in the supportive treatment of sepsis 36,37. At the same time, β-elemene, a phytoestrogen, has anti-inflammatory and endothelial protective effects that are different from those of existing treatments 38. β-elemene has estrogen-like effects and can effectively inhibit the activation of NF-κB, reduce the production of inflammatory factors, and further reduce endothelial damage 39. This gives CW the potential to prevent and treat cardiovascular diseases caused by inflammation, not just sepsis. Future studies should explore the clinical effects of CW and its synergy with existing treatments for different types of vascular injury.
